# Treg cell-derived exosomal miR-21 promotes osteogenic differentiation of periodontal ligament stem cells

**DOI:** 10.1186/s12903-025-06770-0

**Published:** 2025-09-26

**Authors:** Yu Xia, Hao Jiang, Chen Wang, Zhen Liu, Hui Gao, Ji-Feng Yu, Nan Yang, Li Liang

**Affiliations:** 1 Department of Stomatology, Eighth Medical Center of Chinese PLA General Hospital, No. 17, Heishanhu Road, Haidian District, Beijing, 100091 China; 2 Department of Ophthalmology, Beijing Children’s Hospital, Capital Medical University, National Center for Children’s Health, No. 56 Nanlishi Road, Xicheng District, Beijing, 100045 China

**Keywords:** Periodontitis, Regulatory T cells, Exosomes, MicroRNA-21, Osteogenic differentiation

## Abstract

**Background:**

Regulatory T (Treg) cells are essential for maintaining immune tolerance and have been implicated in tissue regeneration; however, their role in periodontal regeneration remains poorly understood. This study aimed to elucidate the effect of Treg cell-derived exosomes (Treg-Exos), particularly those carrying microRNA-21 (miR-21), on the osteogenic differentiation of periodontal ligament stem cells (PDLSCs) and periodontal tissue regeneration.

**Methods:**

After co-culturing Treg cells or Treg-Exos with PDLSCs respectively in vitro, the osteogenic differentiation of treated PDLSCs was assessed through Alkaline phosphatase (ALP) activity, Alizarin Red staining, and the expression of osteogenic markers runt-related transcription factor 2 (Runx-2) and Osterix. The functional role of miR-21 in Treg-Exos was assessed via gain- and loss-of-function experiments, involving transfection of Treg cells with miR-21 mimics or inhibitors. In vivo, a mice periodontal defect model was established via silk ligation and Porphyromonas gingivalis inoculation. Subsequently, Treg-Exos were locally administered into defects to assess their potential in promoting periodontal tissue regeneration. The regenerative efficacy was evaluated using micro-CT and histological analysis.

**Results:**

Treg cell levels were significantly elevated in periodontitis patients compared to healthy controls. Both Treg cells and Treg-Exos markedly promoted the osteogenic differentiation of PDLSCs in vitro, as evidenced by increased ALP activity, enhanced mineralization, and upregulated Runx-2/Osterix expression. Exosomes derived from miR-21-overexpressing Treg cells further promoted PDLSC osteogenesis, whereas exosomes with miR-21 knockdown exhibited an inhibitory effect. In vivo, Treg-Exos injection alleviated periodontal damage and improved tissue morphology compared to PBS controls, as demonstrated by micro-CT and histological analyses.

**Conclusion:**

Treg cell-derived exosomal miR-21 promotes the osteogenic differentiation of PDLSCs, enhancing periodontal tissue regeneration in vivo, suggesting that Treg cells and their exosomal miR-21 may serve as promising therapeutic targets for periodontitis.

**Supplementary Information:**

The online version contains supplementary material available at 10.1186/s12903-025-06770-0.

## Introduction

Periodontitis is a common chronic inflammatory disease that progressively damages periodontal tissues—such as the alveolar bone, cementum, ligament, and gingiva—and, if untreated, leads to tooth loss [1]. This condition is triggered by interactions between pathogenic microorganisms in dental plaque and the host immune response, resulting in chronic inflammation and irreversible tissue degradation [2]. Achieving full periodontal tissue regeneration, particularly of the alveolar bone, remains a primary challenge in therapy despite advances in conventional treatments [3].

Periodontal ligament stem cells (PDLSCs) are a type of mesenchymal stem cell within the periodontal ligament, and they are required in periodontal regeneration because of their self-renewal capabilities and their ability to differentiate into osteoblasts, cementoblasts, or fibroblasts. Enhancing PDLSC osteogenic potential is thus a promising strategy for alveolar bone regeneration and periodontal function restoration [4–6]. However, PDLSC osteogenesis is highly related to the surrounding microenvironment, including immune cells and inflammatory mediators associated with periodontitis [7].

Regulatory T (Treg) cells, a subset of CD4⁺ T cells marked by the transcription factor Foxp3, are responsible for immune tolerance and modulating immune reactions. Beyond immunosuppression, Treg cells take roles in tissue regeneration, such as promoting skeletal muscle recovery and facilitating bone healing in fracture models [8–10]. In bone metabolism, Treg cells can influence osteoclast and osteoblast activity [11–13], though the mechanisms through which they affect PDLSCs and periodontal regeneration remain unclear [14].

Exosomes-nano-sized extracellular vesicles (diameter 30–150 nm) released by various cell types have the significant function of intercellular communication, delivering bioactive molecules like nucleic acids (mRNA and miRNA), proteins, and lipids that modulate recipient cell function [15–21]. Exosomes from Treg cells contribute to immune regulation and have been shown to impact tissue regeneration [22, 23]. Notably, miRNAs in exosomes are capable of post-transcriptional regulation of gene expression in target cells, influencing differentiation, proliferation, and apoptosis. Exosomal miRNAs are important mediators of tissue regeneration and repair [24–28]. MicroRNA-21 (miR-21), a well-known miRNA involved in inflammation, apoptosis, and osteogenesis [29–32], is particularly influential, as it promotes osteogenic differentiation and bone formation [33]. However, the specific role of Treg cell-derived exosomal miR-21 in modulating the osteogenic differentiation of PDLSCs remains unclear.

Therefore, this study was designed to test the hypothesis that Treg cell-derived exosomal miR-21 promotes the osteogenic differentiation of PDLSCs and enhances alveolar bone regeneration. To this end, we first evaluated, in vitro, how Treg cells and Treg cell-derived exosomes (Treg-Exos) affects osteogenic marker expression and functional differentiation assays in PDLSCs. Next, we examined the capacity of Treg-Exos to drive alveolar bone repair in a preclinical periodontitis model, using micro-CT imaging and histological analysis. Finally, gain- and loss-of-function experiments were performed to validate whether Treg-Exos mediate the osteogenic differentiation of PDLSCs through miR-21. Understanding this mechanism may point to new therapeutic targets for the treatment of periodontitis.

## Methods

### Subject design

This work was approved by the Ethics Committee of the Chinese PLA General Hospital (No. 309202406063203). Clinical samples were obtained from the Eighth Medical Center of the PLA General Hospital with informed consent from participants. Female C57BL/6 mice (8–12 weeks old) were sourced from the Laboratory Animal Center at the Eighth Medical Center of Chinese PLA General Hospital (Beijing, China). The work had been reported in line with the ARRIVE guidelines 2.0.

## Sample selection

Human Treg cells were isolated from the blood of 15 healthy participants (control group) and 15 periodontitis patients (periodontitis group), as detailed in Table 1.

**Table 1:** Demographic and clinical characteristics of participants

Group design	*n*	Age^NS^	PD***	Interdental CAL at site of greatest loss	RBL (%)	Tooth loss	RBL/age
Periodontitis (Stage Ⅲ, grade B/C)	15 (9 males, 6 females)	35.7 ± 2.4	6.4 ± 1.2	6.9 ± 1.4	44.2 ± 9.8	1.7 ± 1.4	1.2 ± 0.3
Control (healthy participants)	15 (7 males, 8 females)	33.1 ± 2.6	1.7 ± 0.5	/	/	/	/

The data were presented as mean ± standard deviation (SD)

*CAL* Clinical attachment loss, *RBL* Radiographic bone loss, *PD* Probing depth

^*NS*^ indicated no statistical significance between periodontitis group and control group, ***p < 0.001

The inclusion criteria for control group required the presence of healthy periodontal tissue, as well as the existence of more than one tooth requiring extraction due to orthodontic requirements or dental impaction. Patients with Stage III, Grade B/C periodontitis, as defined by the consensus reports on the new classification system for periodontal diseases and conditions [34], were selected for the test group. Meanwhile, the inclusion criteria for the periodontitis group also required the presence of at least one tooth that was deemed to be extracted as a result of periodontitis-induced severe bone loss or irreversible damage, characterized by evident bleeding upon probing, periodontal pocket depth exceeding 6 mm, tooth mobility of Grade II or higher, and alveolar bone resorption extending to the root apex. To minimize age-related variability, all participants were aged between 30 and 40 years.

The exclusion criteria included pregnancy, lactation, systemic diseases (autoimmune diseases, diabetes), smoking, and immunomodulatory drug use within 3 months of the study.

Control and periodontitis group blood samples were collected from tooth extraction sites. Human peripheral blood was collected from healthy controls for Treg cell isolation.

### Flow cytometry analysis

Peripheral blood mononuclear cells (PBMNCs) were obtained by using Ficoll-Paque Plus (GE, China) by density gradient centrifugation. Cells were stained with CD4, CD25, and CD127 antibodies (eBioscience, USA) and analyzed with a flow cytometer (Beckman, USA). FlowJo software (Tree Star, USA) was used to determine Treg cell proportions in the CD4^*+*^ T cells.

### Cell isolation and culture

PDLSCs were isolated by enzymatic digestion with collagenase type I and dispase (Sigma, USA) and grown in α-MEM containing 10% fetal bovine serum (FBS) and 1% penicillin-streptomycin (Gibco, USA) at 37 °C in 5% CO₂ [35]. Once cell coverage reached about 80%, they were digested by 0.25% trypsin (Beyotime, China) and recorded as primary cells (P0). Cells at passages 2–4 (P2-P4) were used in experiments. Treg cells were purified from healthy participants using the CD4^+^ CD25^+^ Treg Cells Isolation Kit (Miltenyi Biotec, Germany).

### In vitro Co-Culture experiments

In vitro co-culture of Treg cells and PDLSCs was adapted from Liu et al. (2019) [36]. Treg cells and PDLSCs were co-cultured under direct cell-cell contact conditions at a 1:5 ratio in 6-well plates (NEST, China, 10^5^ PDLSCs per well). After 72 h, an osteogenic induction medium (Cyagen Biosciences, China) was used. Alkaline phosphatase (ALP) activity was measured at 14 days by an ALP detection kit (Beyotime, China). Gene and protein expression levels of osteogenesis markers Runt-related transcription factor 2 (Runx-2) and Osterix were assessed by Reverse transcription-polymerase chain reaction (RT-PCR) and Western blot at 7 and 14 days, respectively. Mineralization was assessed by Alizarin Red staining (Sigma) after 21 days, in accordance with the manufacturer’s instructions. Quantification was conducted using a spectrophotometric method. Briefly, mineralized nodules were extracted with 10% cetylpyridinium chloride solution and the absorbance was measured at 562 nm using a microplate reader (BioTek). The data were normalized to total cellular protein content from the same wells, which was quantified using a bicinchoninic acid (BCA) assay kit (Pierce Biotechnology) following cell lysis in 0.1% Triton X-100.

### miRNA-21 expression in exosomes and PDLSCs after Co-culture

*Following a 3-day co-culture of* Treg cells and PDLSCs at a 1:5 ratio, exosomes were isolated from the co-culture supernatants via ultracentrifugation, and PDLSCs were collected by enzymatic digestion. RT-PCR was used to determine the expression levels of miRNA-21 in the exosomes and PDLSCs, with PDLSCs cultured alone as the control.

### Exosome characterization

Treg-Exos were purified by a sequential ultracentrifugation protocol. Culture supernatants were centrifuged (2,000 g, 30 min, 4 ℃), followed by centrifugation at 12,000 g for 45 min to eliminate cell debris, and filtered through a 0.22 μm membrane to remove larger vesicles. Exosome purification was completed with a final ultracentrifugation at 110,000 g for 70 min at 4 ℃. Transmission electron microscopy (TEM, Japan) and nanoparticle tracking analysis (NTA, UK) were used to characterize exosome morphology, diameter distribution, and concentration. Western blotting confirmed exosome markers (CD63 and TSG101).

### Co-culture of Treg-Exos and PDLSCs

PDLSCs were put in 6-well plates (1 × 10^5^ cells/well) to reach ~ 80% confluency. Treg cell-conditioned medium (TCM), containing 50 µg exosomes per 1 ml cell culture medium, was used to co-culture PDLSCs for 3 days in the Tregs Exo group. Alone PDLSCs in an equivalent cell culture medium served as the control group. Then, PDLSCs were grown in an osteogenic induction complete medium (Cyagen Biosciences Inc). Osteogenic differentiation of PDLSCs was then assessed using ALP activity detection at day 14, Alizarin Red staining at day 21, and expression levels of osteogenesis markers Runx-2 and Osterix at days 7 and 14.

### Overexpression and knockdown of miRNA-21 in Treg cells

Treg cells were transfected with miR-21 mimics (pre-miR-21), inhibitors (anti-miR-21), or respective negative controls (pre-miR-21-cont and anti-miR-21-cont) using Lipofectamine 2000 (Ambion, Austin, TX) according to the manual. Transfection efficiency was confirmed by RT-PCR analysis. Exosomes were isolated from transfected Treg cells, and miRNA-21 levels were evaluated. Exosomes were subsequently added to PDLSCs and cultured for 3 days, after which miRNA-21 presence and PDLSCs’ osteogenic differentiation (assessed via ALP activity, Alizarin Red staining, and Runx-2 and Osterix expression) were measured.

### RT-PCR analysis

Total RNA was extracted with TRIzol reagent (Invitrogen, USA), and reverse transcription was performed using PrimeScript RT (TaKaRa, Japan). RT-PCR primers specific to miR-21 and the internal control U6 (Riobobio) were used for miRNA. Primer sequences for RT-PCR are provided in Table 2. β-actin as a control for protein-encoding genes. Amplification was conducted using the SYBR Premix Ex Taq II kit (TaKaRa), and detection on the Applied Biosystems ABI Prism 7500 HT sequence detection system (Applied Biosystems, Germany).

**Table 2:** Human primers sequences for RT-PCR

Gene	Forward Primer	Reverse Primer
Runx-2	5’-CACAAGTGCGGTGCAAACTT-3’	5’-TGCTTGCAGCCTTAAATGACT-3’
Osterix	5’-GAGAGGAGAGACTCGGGACA-3’	5’-AGTGAACTTCCTCCTCAAGCA-3’
β-actin	5’- CATGTACGTTGCTATCCAGGC − 3’	5’- CTCCTTAATGTCACGCACGAT − 3’

### Western blotting

Proteins from PDLSCs, Treg cells, and exosomes were isolated with cell lysis buffer (Beyotime) and quantified by BCA assay (Pierce™). Protein samples were loaded on SDS-PAGE (Sigma), transferred to polyvinylidene fluoride (PVDF) membranes (Millipore, USA), and incubated with the primary antibodies anti-Runx-2 and anti-Osterix (Affinity Biosciences, USA), anti-β-actin, anti-CD63, and anti-TSG101 (Abcam). This was followed by anti-horseradish peroxidase-conjugated secondary antibodies (Abcam).

### In vivo mice periodontal defect model

Female C57BL/6 mice were randomly allocated into two groups at a 1:1 ratio. The sample size was determined based on a pilot study measuring cemento-enamel junction to the alveolar bone crest (CEJ-ABC) distance (*n* = 3 per group), a power analysis (two-sided test, α = 0.05, power = 80%) indicated a minimum requirement of 6 mice per group. To account for potential exclusions (e.g., ligature shedding, mortality), 7 mice were allocated per group.

Periodontal defects were established through silk ligation and bacterial inoculation [37, 38]. Under anesthesia (pentobarbital sodium, 40 mg/kg), a 5 − 0 silk ligature was placed into the interproximal space between the maxillary right first and second molars and tightened to induce subgingival retention. Control mice received anesthesia without ligation. Gingiva at the ligature site were coated with Porphyromonas gingivalis (Pg ATCC 33277, American Type Culture Collection, USA) four times per week for one month. Control mice were coated with PBS.

One month post-induction, mice were euthanized via cervical dislocation. Maxillae were harvested, and periodontal defects were validated by micro-CT, H&E staining, and Masson staining. Six mice per group were randomly selected for final analysis.

### Injection and tracking of Treg-Exos in vivo

For exosome treatment, mice were administered Treg-Exos twice weekly for a duration of three weeks using an insulin syringe. Control mice were administered an equal volume of PBS. After a 21-day intervention with exosomes or PBS, mice were euthanized. Subsequently, exosomal membranes were labeled using anti-human DiL (SCR-Biotech, Shanghai, China), while nuclei were stained with anti-mice DAPI (C1006, Beyotime, Shanghai, China). Fluorescence microscopy (Olympus, Tokyo, Japan) was utilized to visualize exosomes surrounding the periodontal defect area. The obtained periodontal defects were further analyzed using Micro CT, H&E staining, and Masson staining.

### Micro-CT and histological analysis

Mice maxillary bone and teeth were fixed with 4% paraformaldehyde (Sangon Biotech, China) for more than 24 h and then examined using a Viva CT 40 scanner (SCANCO Medical, Switzerland) to assess bone integrity and structural changes in the periodontal defect area. Periodontal tissues were further decalcified with 10% EDTA (pH 7.0), embedded in paraffin, and sectioned into 5-µm-thick slices. Histological evaluations were conducted using the Hematoxylin and Eosin (H&E) Stain Kit (G1120, Solarbio, China) and Masson Staining Kit (DC0032, Leagene, China).

### Statistical analysis

All data were presented as mean ± standard deviation (SD). Normality was assessed using the Shapiro-Wilk test. For intergroup comparisons between two groups, Student’s t-test or Mann-Whitney U test was applied depending on data distribution. For comparisons involving more than two groups, ANOVA or Kruskal-Wallis test was applied as appropriate. All statistical analyses were conducted using GraphPad Prism 9.0 software. Two-sided tests were performed, and *p* < 0.05 was considered statistically significant.

## Results

### Increased Treg cells in periodontitis and their enhancement of PDLSC osteogenic differentiation in vitro

**Fig. 1:**
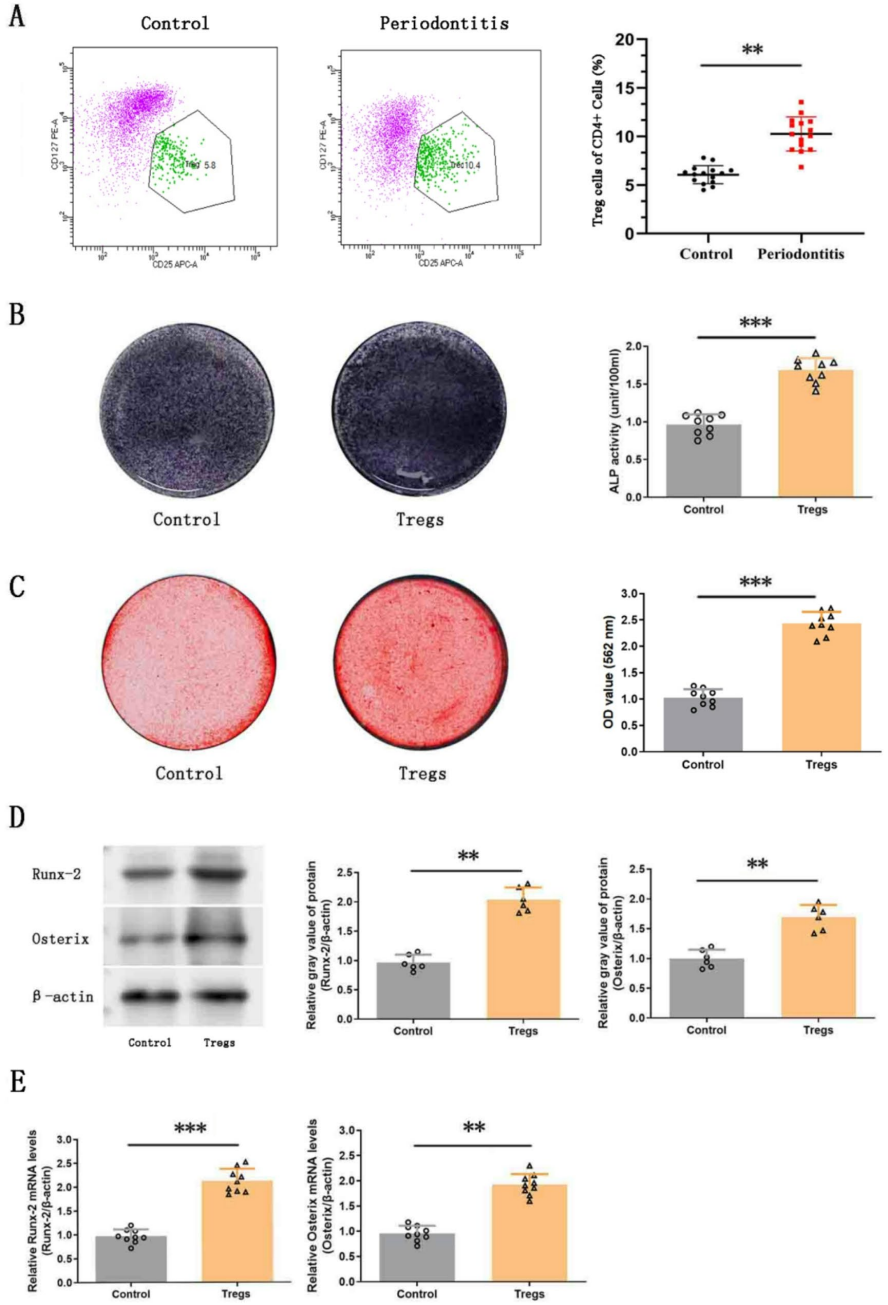
Osteogenic differentiation of human periodontal ligament stem cells (PDLSCs) was significantly enhanced following in vitro co-culture with human regulatory T cells (Tregs group) at a ratio of 1:5 (Treg cells: PDLSCs). **A** Treg cells were identified and purified via flow cytometry. Treg cell expression levels in periodontitis patient blood samples were significantly higher than those in healthy participants. **B** Alkaline phosphatase (ALP) and (**C**) Alizarin Red staining of PDLSCs after 14-day and 21-day osteogenic induction. **D** and **E** Expression levels of osteogenic-associated proteins and genes (Runx-2 and Osterix) were measured by Western blot and RT-PCR analysis. Full-length blots/gels are presented in Supplementary Fig. 1. Data are presented as mean ± SD; *n* = 15 (in A), 9 (in B, C, and E), and 6 (in D). **p < 0.01 and ***p < 0.001

Flow cytometry analysis revealed a significant increase in Treg cell levels in the periodontal blood of periodontitis patients compared to healthy controls (*p* < 0.01; Fig. 1A).

To explore the effect of Treg cells on the osteogenic differentiation of PDLSCs, in vitro co-culture experiments were conducted. ALP activity and bone nodule formation were markedly enhanced in the Tregs group, as shown by ALP and Alizarin Red staining (*p* < 0.001; Figs. 1B and C). Additionally, Western Blot and RT-PCR analyses showed significantly elevated expression of osteogenic markers Runx-2 and Osterix in the Tregs group in comparison to controls (*p* < 0.01 or 0.001; Figs. 1D and E), indicating that Treg cells promote PDLSC osteogenic differentiation in vitro.

### Promotion of PDLSC osteogenic differentiation by Treg-Exos in vitro

**Fig. 2:**
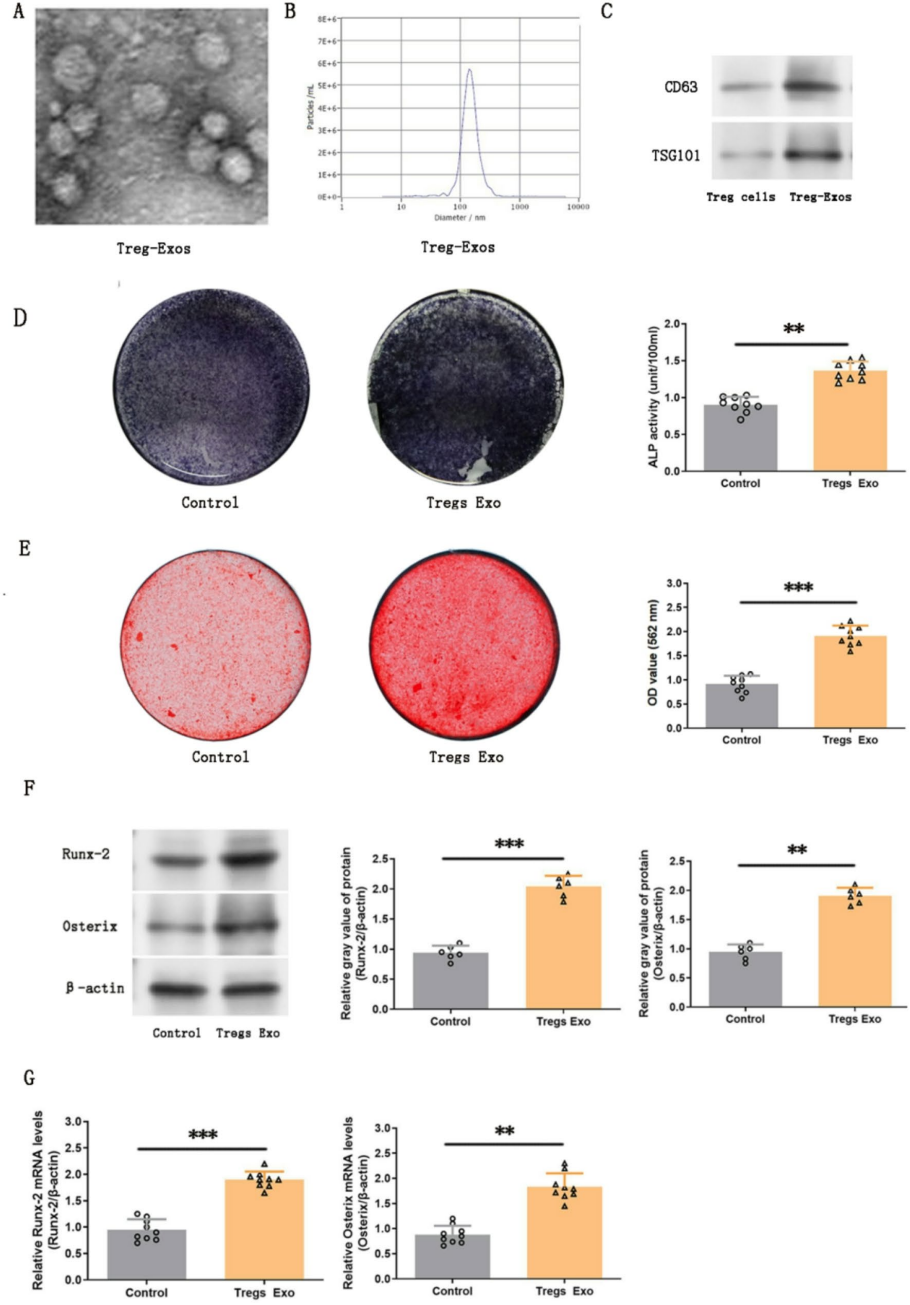
Osteogenic differentiation of human PDLSCs was significantly enhanced following in vitro co-culture with human Treg cell-derived exosomes (Tregs Exo group). **A** Electron microscope image of Treg cell-derived exosomes (Treg-Exos). **B** Exosome density and diameter analysis. **C** Western blot analysis of exosomal surface markers, including CD63 and TSG101. Full-length blots/gels are presented in Supplementary Fig. 2. **D** ALP and (**E**) Alizarin Red staining of PDLSCs after 14-day and 21-day osteogenic induction. **F** and **G** Expression levels of osteogenic-associated proteins and genes (Runx-2 and Osterix) were assessed by Western blot and RT-PCR. Full-length blots/gels are presented in Supplementary Fig. 3. Data are presented as mean ± SD; *n* = 9 (in **D**, **E**, and **G**) and 6 (in **F**). **p < 0.01, and ***p < 0.001

To assess the role of exosomes in PDLSC osteogenic differentiation, Treg-Exos were isolated from Treg cell culture supernatants and verified by TEM, NTA, and Western blot. TEM revealed nanoparticles 50–150 nm in diameter, and NTA analysis confirmed a size distribution (Figs. 2A and B). Exosomal markers CD63, and TSG101 were detected by Western blot (Fig. 2 C), confirming successful exosome separation. PDLSCs were co-cultured with TCM for 3 days, followed by osteogenic induction. After 14 days, ALP activity was significantly increased in Treg-Exos-treated PDLSCs (*p* < 0.01; Fig. 2D). By day 21, mineralized nodule formation was markedly enhanced (*p* < 0.001; Fig. 2E). Consistent with functional assays, RT-PCR and Western blot analyses revealed significant upregulation of osteogenic markers Runx-2 and Osterix at both gene and protein levels (*p* < 0.01, or *p* < 0.001; 2 F and G). These results suggest that Treg-Exos enhance osteogenic differentiation of PDLSCs in vitro.

### Construction of periodontal defect model in mice

**Fig. 3:**
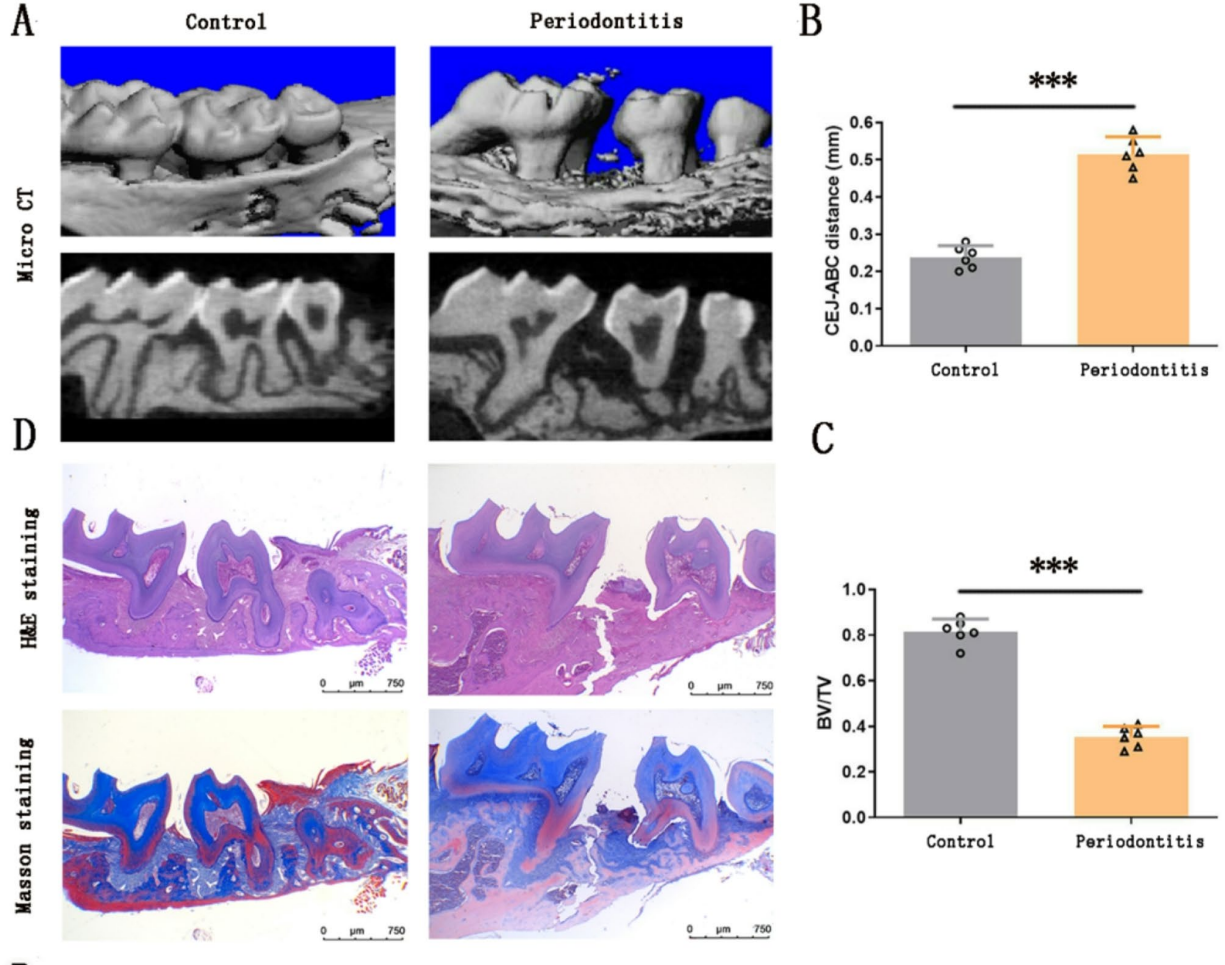
Construction and validation of a C57BL/6 mice periodontal defect model. **A** Micro-CT of the periodontal defect model. **B** and **C** Measurements of the distance from the cemento-enamel junction to the alveolar bone crest (CEJ-ABC) and the bone volume to tissue volume (BV/TV) ratio, as quantified analysis by Micro-CT data. **D** H&E staining and Masson staining of the periodontal defect model. Data are presented as mean ± SD; *n* = 6. ***p < 0.001

A periodontal defect model was established in C57BL/6 mice by ligating the molars with silk thread and applying *Porphyromonas gingivalis* to the gingiva. Untreated mice served as controls. After one month, alveolar bone destruction was assessed. Micro-CT quantitative analysis revealed significantly increased CEJ-ABC distance and decreased bone volume to tissue volume ratio (BV/TV) in the periodontitis group compared to controls (*p* < 0.001; Figs. 3A-C). Histological assessment using H&E staining and Masson staining provided qualitative evidence of alveolar bone loss in the periodontitis group, which was consistent with the Micro-CT findings (Fig. 3D). These results collectively indicate the successful construction of a periodontal defect model.

### Treg-Exos mitigate periodontal damage and promote bone regeneration in vivo

**Fig. 4:**
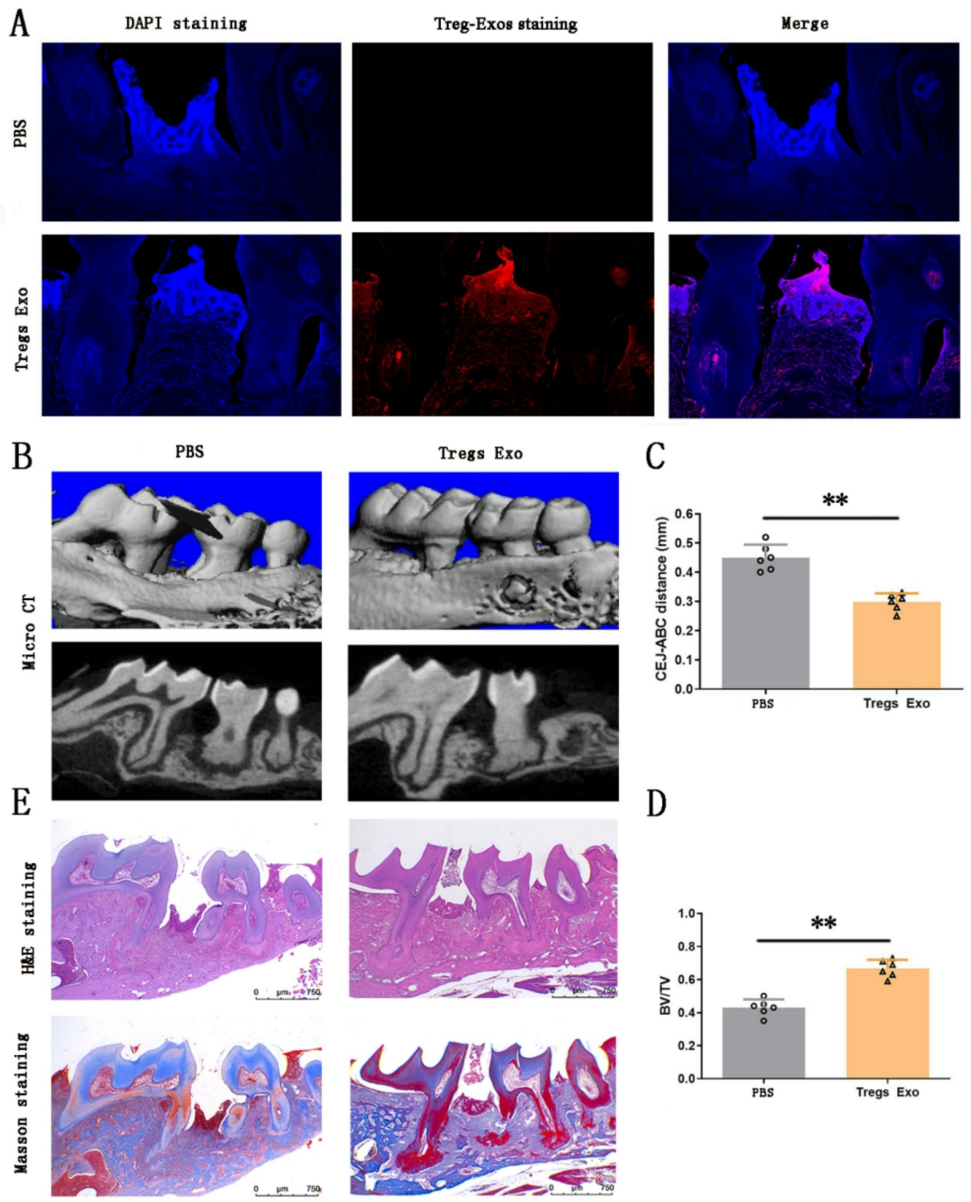
Local injection of Treg-Exos significantly promotes bone tissue repair in the mice periodontal defect area. **A** Exosome distribution was detected in the periodontal defect area by laser confocal microscopy (nucleus, blue; exosomes, red). **B** Micro-CT of the periodontal defect model. **C** CEJ-ABC distance and (**D**) BV/TV ratio indicate that exosomes can significantly promote periodontal tissue regeneration in vivo, as quantified analysis by Micro-CT data. **E** H&E and Masson staining of the periodontal defect area. Data are presented as mean ± SD; *n* = 6. **p < 0.01

Following model validation, Treg-Exos was injected into the periodontal defect area in the Tregs Exo group, while PBS was administered to the control group. Immunofluorescence analysis confirmed exosome location in the defect area (Fig. 4A). Micro-CT quantitative analyses revealed a significant reduction in the CEJ-ABC distance and higher BV/TV in the Tregs Exo group compared to PBS controls (*p* < 0.01; Figs. 4B-D). Qualitative histological assessment using H&E staining and Masson staining further supported these findings, showing reduced alveolar bone loss and improved tissue morphology in the Tregs Exo group (Fig. 4E). These findings collectively suggest that Treg-Exos mitigate periodontal damage and facilitate bone regeneration.

### Exosomes may transfer miR‑21 to PDLSCs

**Fig. 5:**
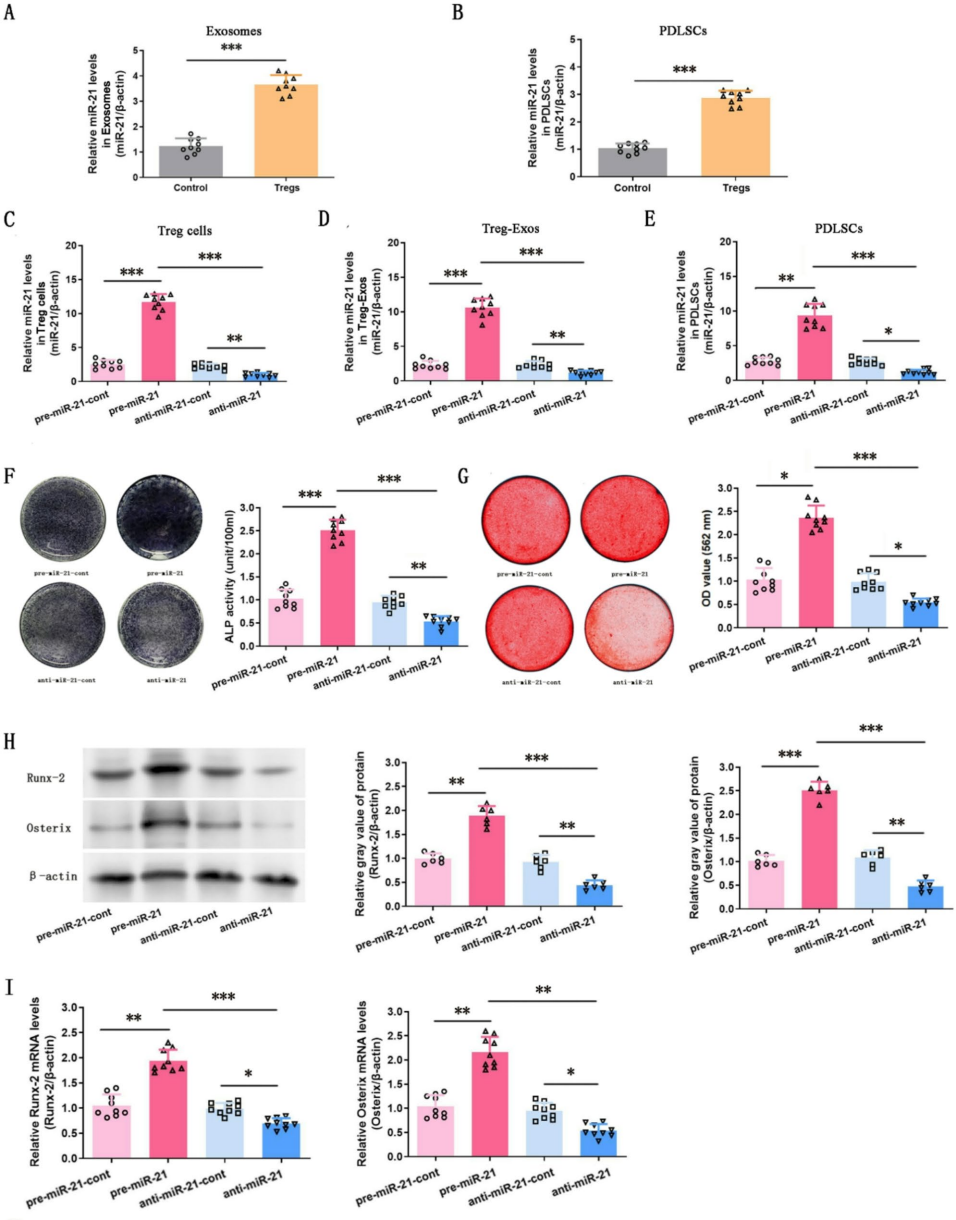
Elevated expression of miR-21 in Treg-Exos enhances osteogenic differentiation of PDLSCs in vitro. **A** Relative expression of miR-21 in exosomes derived from the co-culture supernatants. **B** Relative expression of miR-21 in Treg cell-treated PDLSCs. **C**-**E** Treg cells with miR-21 overexpression (pre-miR-21) and knockdown (anti-miR-21) were generated, and transfection efficiency was assessed in Treg cells, Treg-Exos, and Treg cell-treated PDLSCs via RT-PCR. Exosomes derived from pre-miR-21 control Treg cells, pre-miR-21 Treg cells, anti-miR-21 control Treg cells, and anti-miR-21 Treg cells were co-cultured with PDLSCs for 3 days. **F** and **G** ALP and Alizarin Red staining after 14-day and 21-day osteogenic induction indicated that osteoblastic differentiation was significantly enhanced in the pre-miR-21 group but reduced in the anti-miR-21 group. (H and I) Western blot and RT-PCR analyses for Runx-2 and Osterix expression levels. Full-length blots/gels are presented in Supplementary Fig. 4. The results indicate the enhanced osteogenic differentiation ability in the pre-miR-21 group and reduced in the anti-miR-21 group, suggesting miR-21’s involvement in osteogenesis of PDLSCs. Data are presented as mean ± SD; *n* = 9 (in A-G and I) and 6 (in H). *p < 0.05, **p < 0.01, and ***p < 0.001

Exosomes are known to transfer miRNAs, which modulate biological processes. RT-PCR analysis revealed a significant increase of miR-21 levels in exosomes derived from the co-culture supernatants (*p* < 0.001; Fig. 5A). Additionally, miR-21 expression in Treg cell-treated PDLSCs was significantly elevated, suggesting that exosomes may transfer miR‑21 from Treg cells to PDLSCs (*p* < 0.001; Fig. 5B).

### Treg-Exos enhanced the osteogenic differentiation of PDLSCs in vitro by delivering miR-21 to PDLSCs

To investigate exosomal miR-21’s function in Treg-Exos-mediated osteogenesis, lentiviral transfection generated Treg cells overexpressing (pre-miR-21) or knocking down (anti-miR-21) miR-21, along with negative controls (pre-miR-21-cont and anti-miR-21-cont). Transfection efficiency was verified by RT-PCR (*p* < 0.01 or *p* < 0.001; Fig. 5C).

Exosomes were then extracted from transfected Treg cells (pre-miR-21-cont-Exos, pre-miR-21-Exos, anti-miR-21-cont-Exos, and anti-miR-21-Exos) and co-cultured with PDLSCs. miR-21 was significantly altered in PDLSCs depending on the exosome type, aligning with the corresponding group (*p* < 0.05, *p* < 0.01, or *p* < 0.001; Fig. 5D and E). To evaluate osteogenic differentiation, the four types of Treg-Exos were co-cultured with PDLSCs for 3 days and then induced into osteogenesis. Following 14 and 21 days of induction, ALP and Alizarin Red staining indicated the highest osteogenic differentiation in the pre-miR-21 group and significantly reduced differentiation in the anti-miR-21 group (*p* < 0.05, *p* < 0.01, or *p* < 0.001; Figs. 5F and G). Runx-2 and Osterix expression levels were likewise enhanced in the pre-miR-21 group, while the anti-miR-21 group showed decreases (*p* < 0.05, *p* < 0.01, or *p* < 0.001; Figs. 5H and I).

## Discussion

**Fig. 6:**
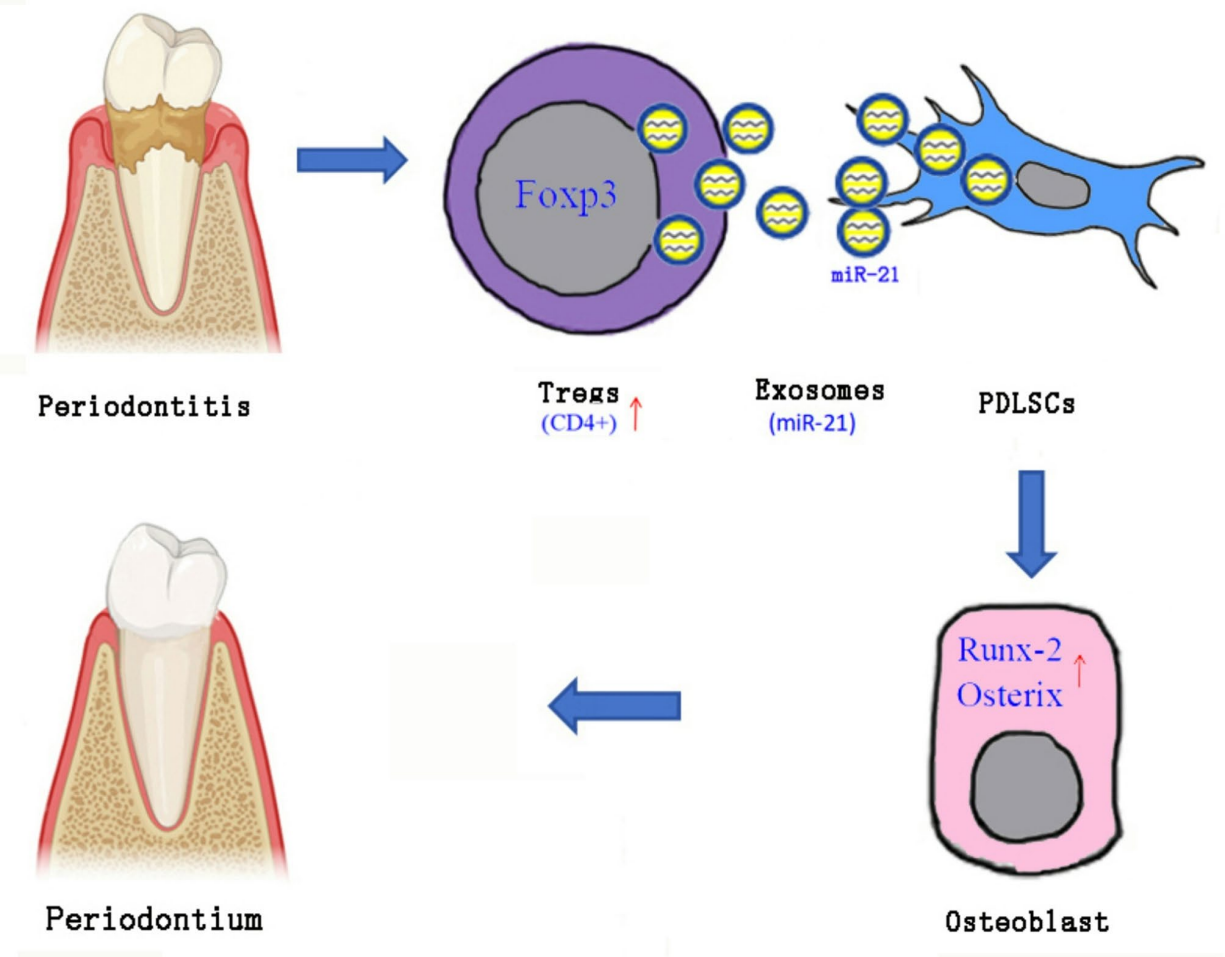
Graphical abstract of the study. Treg cell-derived exosomal miR-21 promotes osteogenesis of PDLSCs. miR-21: microRNA-21, PDLSCs: periodontal ligament stem cells

In this study, we employed both in vitro and in vivo methods to examine the role of Treg cells and their exosome-derived miR-21 in promoting the osteogenic differentiation of PDLSCs and facilitating periodontal tissue regeneration. Treg cells enhance PDLSC osteogenesis by secreting exosomal miR-21. Injection of Treg-Exos in a mice periodontal defect model significantly mitigated periodontal damage and promoted bone regeneration. These findings suggest that Treg cells and their exosomal miR-21 offer promising therapeutic strategies for periodontal regeneration in periodontitis (Graphical abstract of the study, Fig. 6).

Periodontitis, a chronic inflammatory disease characterized by periodontal tissue degradation and subsequent alveolar bone loss, necessitates effective restoration strategies. PDLSCs are key to periodontal regeneration due to their osteogenic potential [4–6]. Previous research has demonstrated that modulating the immune microenvironment can significantly influence stem cell-mediated tissue regeneration [7]. While Treg cells are recognized for their immunosuppressive role in immune homeostasis [39–42], their influence on PDLSC osteogenesis and periodontal regeneration has remained unclear until now.

Flow cytometry analysis indicated that Treg cell levels in periodontal blood were significantly higher in periodontitis patients than in healthy controls, suggesting a role in disease pathogenesis or repair processes. This observation is consistent with previous studies showing elevated Treg cell populations in various inflammatory and regenerative contexts [43–45]. In vitro co-culture experiments further demonstrated that Treg cells enhance PDLSC osteogenesis, evidenced by increased ALP activity, bone nodule formation, and upregulated expression of osteogenic markers Runx-2 and Osterix, indicating that Treg cells support osteogenic differentiation of PDLSCs.

Exosomes, nano-sized extracellular vesicles, are integral to intercellular communication by transferring genetic material, including miRNAs, to regulate cellular functions [15–21]. To explore the mechanisms by which Treg cells modulate PDLSCs, we hypothesized that Treg-Exos mediate PDLSC osteogenesis. Exosomes isolated from Treg cell culture supernatants were characterized by TEM, NTA, and Western blotting for CD63 and TSG101 markers. Co-culture with Treg-Exos significantly promoted PDLSCs osteogenesis, indicated by enhanced ALP activity, mineralization, and upregulation of osteogenic markers. These results suggest that Treg-Exos can effectively promote the osteogenic differentiation of PDLSCs.

In vivo experiments further substantiated the therapeutic potential of Treg-Exos. Administration of these exosomes in a mice periodontal defect model significantly reduced the CEJ-ABC distance and increased BV/TV, indicating that Treg-Exos mitigated periodontal damage and promoted bone regeneration.

Exosomal miRNAs were previously reported to regulate recipient cell functions [24–28]. The elevated level of miR-21 in Treg cell-treated PDLSCs suggested that miR-21 may be transferred from Treg cells to PDLSCs via exosomes. Known for its roles in cell proliferation, differentiation, and bone metabolism, miR-21 was further investigated as a potential mediator of PDLSC osteogenesis. This aligns with studies that have identified miR-21 as a pivotal regulator in various osteogenic and regenerative processes [30–32].

To further investigate the effects of miR-21 derived from Treg cells on the osteogenic potential of PDLSCs in vitro, we conducted comprehensive functional analyses through both gain- and loss-of-function studies. We manipulated miR-21 expression in Treg cells using lentiviral vectors to overexpress or knockdown miR-21. Exosomes from these modified Treg cells were co-cultured with PDLSCs, revealing that overexpression significantly enhanced osteogenesis, while knockdown reduced these effects. These findings confirm that miR-21 in Treg-Exos is a key mediator in promoting PDLSC osteogenesis. This advancement provides a deeper understanding of the molecular mechanisms by which immune cells influence stem cell differentiation.

The majority of literature reports [33, 46, 47], in conjunction with our previously published findings [48], indicated that miR-21 acted as a critical promoting factor for the osteogenic differentiation of bone marrow mesenchymal stem cells and PDLSCs. However, other studies have proposed that miR-21 may promote osteoclastogenesis and differentiation [49, 50]. This functional versatility implies that miR-21 exerts distinct regulatory effects under varying physiological and pathological settings, suggesting a potentially significant regulatory role in bone metabolism. miR-21 appears involved not only in bone formation but also potentially associated with mechanisms underlying bone resorption, thereby modulating bone remodeling. The pro-osteogenic effect observed in our study (mediated by Treg-Exos), alongside its reported inhibitory potential, supports this dual functionality. This indicates that the net effect of miR-21 is highly context-dependent, influenced by factors such as its cellular source (e.g., Treg cells), mode of delivery (exosomal vs. synthetic), and the specific microenvironment (e.g., inflammatory status or regenerative phase). These findings further underscore the pivotal role of miR-21 in maintaining bone balance and homeostasis, while its precise mechanism warrants further in-depth investigation.

Despite these promising findings, this study has several limitations. First, the in vitro co-culture model, while informative, does not fully replicate the complex in vivo periodontal microenvironment. Second, the mice model, although widely used, may not entirely capture the intricacies of human periodontitis and periodontal regeneration. Third, the comprehensive molecular profiling of Treg-Exos (e.g., proteomics, lipidomics, full miRNA sequencing) was not performed in this study. Future studies will employ multi-omics approaches to identify additional bioactive components contributing to periodontal regeneration.

Collectively, our findings demonstrate that Treg cell-derived exosomal miR-21 acts as a key molecular signal enhancing PDLSC osteogenesis and alveolar bone regeneration. This mechanism reveals a dual role for Treg cells in periodontal homeostasis, extending their function beyond immunosuppression to active participation in tissue repair. Furthermore, the ability of exogenous Treg-Exos to reduce bone loss in vivo demonstrates their potential as a therapy for periodontal tissue regeneration.

## Conclusions

In summary, this study demonstrates that Treg cell-derived exosomal miR-21 serves as a critical mediator in promoting the osteogenic differentiation of PDLSCs and enhancing alveolar bone regeneration. These findings elucidate a novel immune-stem cell crosstalk mechanism mediated by Treg cell exosomal miR-21, highlighting its potential as a promising therapeutic strategy for periodontal regeneration.

## Supplementary Information

Supplementary Material 1.

## Data Availability

No datasets were generated or analysed during the current study.

## Abbreviations

ALP: Alkaline phosphatase
DAPI: 4’,6-Diamidino-2-phenylindole
H&E staining: Hematoxylin and eosin staining
miR-21: MicroRNA-21
PBMNCs: Peripheral blood mononuclear cells
PDLSCs: Periodontal ligament stem cells
Pg: Porphyromonas gingivalis
RT-PCR: Reverse transcription-polymerase chain reaction
Runx-2: Runt-related transcription factor 2
TCM: Treg cell-conditioned medium
TEM: Transmission electron microscopy
Treg cells: Regulatory T cells
Treg-Exos: Treg cell-derived exosomes
