# Supplementary material for: Treg cell-derived exosomal miR-21 promotes osteogenic differentiation of periodontal ligament stem cells
Source: BMC Oral Health. 2025 Sep 26;25:1465. doi: 10.1186/s12903-025-06770-0 (PMC12465503; doi:10.1186/s12903-025-06770-0)

Treg Cell-Derived Exosomal miR-21 Promotes Osteogenic Differentiation of Periodontal Ligament Stem Cells

Yu Xia[1†], Hao Jiang[1†], Chen Wang[1†], Zhen Liu[1], Hui Gao[1], Ji-Feng Yu[2*], Nan Yang[1*] and Li Liang[1*]

Supplementary figure 1. The original, unprocessed full-length gels and blots of the gel/blot images in Figure 1D.

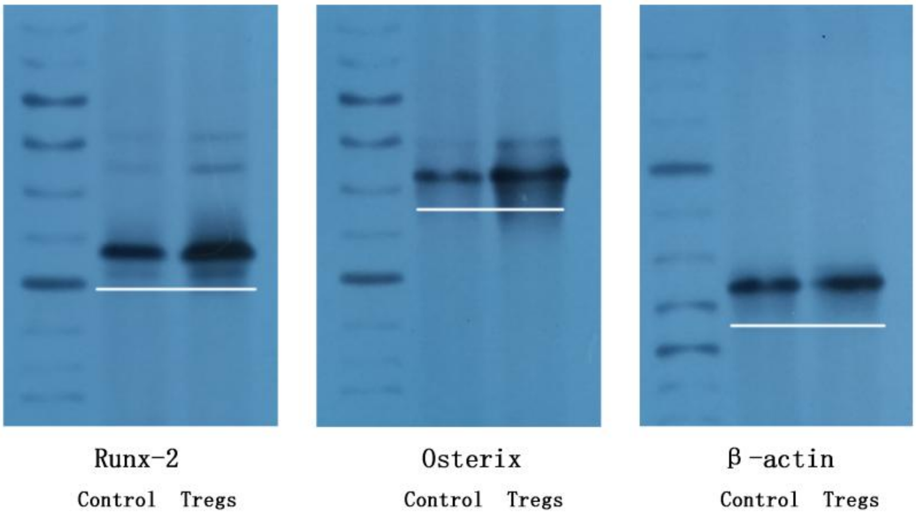

Supplementary figure 2. The original, unprocessed full-length gels and blots of the gel/blot images in Figure 2C.

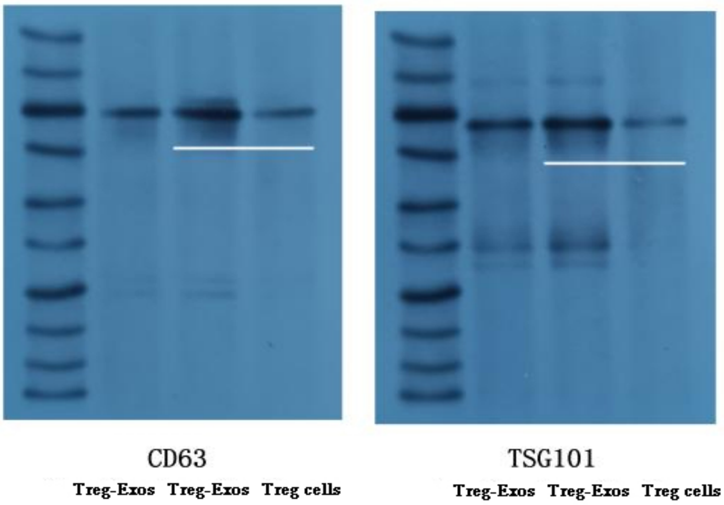

Supplementary figure 3. The original, unprocessed full-length gels and blots of the gel/blot images in Figure 2F.

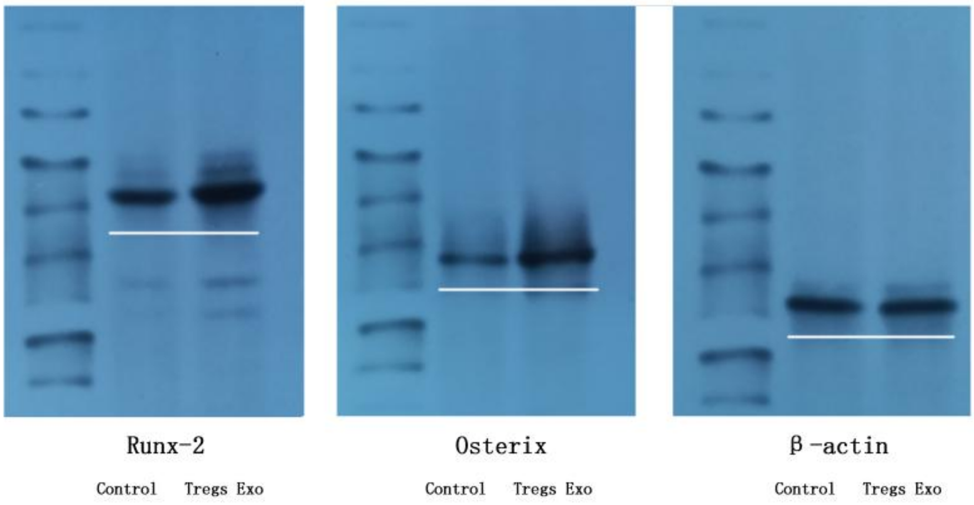

Supplementary figure 4. The original, unprocessed full-length gels and blots of the gel/blot images in Figure 5H.

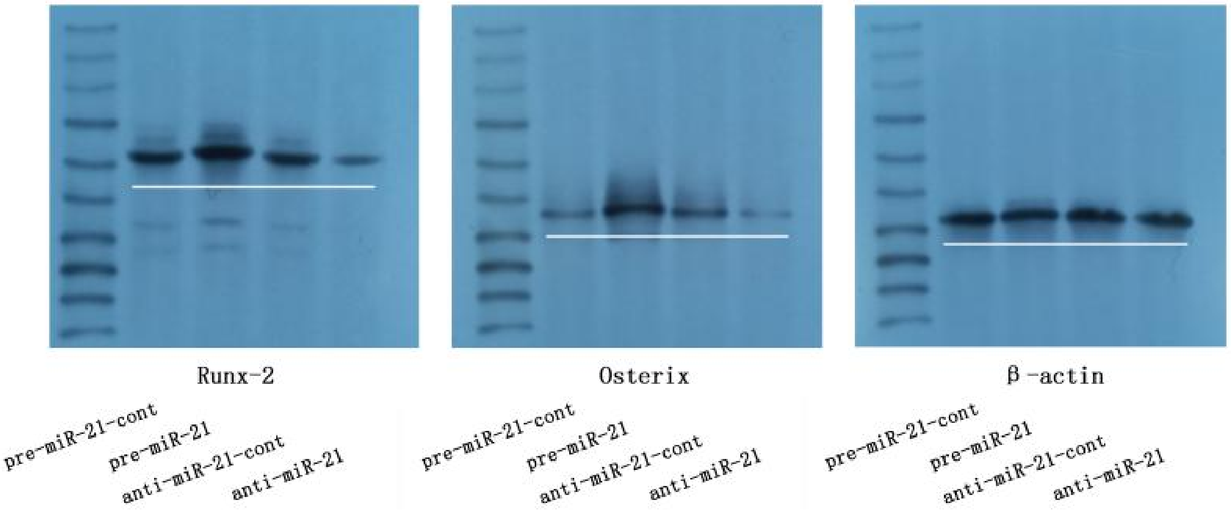

Supplement: Supplementary file 1 — Supplementary Material 1. [file 12903_2025_6770_MOESM1_ESM.pdf]
